# Parental Autonomy in the Care of Premature Newborns and the Experience of a Neonatal Team: Observational Prospective Study

**DOI:** 10.2196/55411

**Published:** 2024-08-30

**Authors:** Salvador Piris-Borregas, Beatriz Bellón-Vaquerizo, Leticia Velasco-Echeburúa, Lidia Niño-Díaz, Susana Sánchez-Aparicio, María López-Maestro, Carmen Rosa Pallás-Alonso

**Affiliations:** 1 Department of Neonatology, Instituto de Investigación Biomédica, 12 de Octubre University Hospital, Avenida de Córdoba s/n, Madrid, 28041, Spain, 34 91390195

**Keywords:** family-centered care, neonatal intensive care unit, kangaroo mother care, mother, mothers, parent, parents, parental, ICU, intensive care, training, education, educational, premature, pediatric, pediatrics, paediatric, paediatrics, infant, infants, infancy, baby, babies, neonate, neonates, neonatal, newborn, newborns, intensive care unit

## Abstract

**Background:**

The European Foundation for the Care of Newborn Infants (EFCNI) has promoted the importance of parental involvement in the care of children.

**Objective:**

The study aimed to examine how the time required by parents to achieve autonomy in the care of their very low–birth weight newborn infants was modified during the implementation of a training program.

**Methods:**

This was an observational prospective study in the context of a quality improvement initiative. The Cuídame (meaning “Take Care of Me” in English) program was aimed at achieving parental autonomy. It was implemented over 2 periods: period 1, from September 1, 2020, to June 15, 2021; and period 2, from July 15, 2021, to May 31, 2022. The days required by parents to achieve autonomy in several areas of care were collected from the electronic health system.

**Results:**

A total of 54 and 43 families with newborn infants were recruited in periods 1 and 2, respectively. Less time was required to acheive autonomy in period 2 for participation in clinical rounds (median 10.5, IQR 5‐20 vs 7, IQR 4‐10.5 d; *P*<.001), feeding (median 53.5, IQR 34‐68 vs 44.5, IQR 37‐62 d; *P*=.049), and observation of neurobehavior (median 18, IQR 9‐33 vs 11, IQR 7‐16 d; *P*=.049). More time was required to achieve autonomy for kangaroo mother care (median 14, IQR 7‐23 vs 21, IQR 10‐31 d; *P*=.02), diaper change (median 9.5, IQR 4‐20 vs 14.5, IQR 9‐32 d; *P*=.04), and infection prevention (median 1, IQR 1‐2 vs 6, IQR 3‐12; *P*<.001).

**Conclusions:**

Parents required less time to achieve autonomy for participation in clinical rounds, feeding, and observation of neurobehavior during the implementation of the training program. Nevertheless, they required more time to achieve autonomy for kangaroo mother care, diaper change, and infection prevention.

## Introduction

The European Foundation for the Care of Newborn Infants (EFCNI) has promoted the importance of parental involvement in the care of children [1]. It has been expected that parental autonomy will result in additional benefits. Some programs have been created [2-4] to achieve such training. However, the time required by parents to become autonomous is unknown, particularly in the case of premature children.

The main objective of this study is to compare the number of days required by parents of very low–birth weight (VLBW) newborn infants to be autonomous in different areas of care during the 2 periods of implementation of a parental training program.

## Methods

### Overview

This observational prospective study was associated with a quality improvement initiative, aimed at increasing parental autonomy in the care of VLBW newborn infants during the implementation of the Cuídame (meaning “Take Care of Me” in English) program in a IIIC-level Spanish neonatal unit.

Prior to the implementation of this program, in 2020, materials were prepared, and meetings with the most sensitive health care providers to neurodevelopmental-centered care issues were conducted. The program was implemented over 2 periods (Table 1). Period 1 was from September 1, 2020, to June 15, 2021, representing the start of the implementation of the Cuídame program. Period 2 was from July 15, 2021, to May 31, 2022, representing complete implementation along with greater experience of the neonatal team.

**Table 1.** Actions carried out in the neonatal unit aimed at the implementation of the Cuídame program over time, as well as the events that could act as barriers to the implementation.

Periods	Actions	Barriers
Prior to the implementation	The use of family rooms had already begun Design of the Cuídame program Approval by the Ethics Committee Selection of professionals responsible for monitoring the program Presentation of the program to the professionals	Family members are restricted due to the COVID-19 epidemic
Period 1: from September 1, 2020, to June 15, 2021	Start of the implementation of the Cuídame program	Family members are restricted due to the COVID-19 epidemic
Period 2: from July 15, 2021, to May 31, 2022	Complete implementation of the Cuídame program	Family members are restricted due to the COVID-19 epidemic Summer vacation Incorporation of new professionals

This program included several areas of care and a paper called a road map, where the parents were encouraged to write down the dates they achieved autonomy under the supervision of the neonatal nursing staff [5]. Health professionals provided information about the Cuídame program at a time close to the admission. There was no standardized day to initiate the information provision. The team adapted to the individual situation of each child and family. A responsible professional was assigned to each family to monitor the autonomy of the parents in caring for their babies. In addition, professionals received file books with the criteria and indicators that parents had to meet to advance in every step.

The families who recorded the dates of autonomy in all areas of care on their road maps were included. Autonomy scores were not based on whether the primary caregiver was the mother or the father. In the context of clinical management, the families of newborns infants with a severe disease focused on caring for children at the end of their life, families with newborn infants who died during the first month of life, or those with important language barriers were excluded.

The autonomy of parents in caring for their VLBW newborn infants during their admission to the neonatal unit and the dependent variables related to the morbidity of VLBW newborn infants were defined based on scientific literature [5]. Autonomy in kangaroo mother care was achieved when parents transferred newborn infants from the incubator to their chest, placed their children properly in the kangaroo position, and then transferred the children back to the incubator. With respect to feeding, autonomy was achieved when parents were able to recognize the signs that their infants were hungry and feed them orally. Autonomy in the observation of neurobehavior was achieved when parents identified their infants’ daily achievements and helped them reach the next neurobehavioral step. The parents were considered autonomous in handling, posture, and contact when they created the cradle nest, chose the bedding, placed the infants properly, and executed postural changes in the incubator. Autonomy in diaper change occurred when the infants’ diaper were changed by the parents themselves. When parents detected their infants’ pain or stress signs and applied nonpharmacological analgesic measures, they were considered autonomous in stress and pain prevention. Autonomy in the prevention of sensory deprivation was achieved when the parents talked to their infants, chose stories to tell them, and conveyed the importance of this initiative to other parents. Autonomy in patient safety involved ensuring that the parents dealt with their babies, were familiar with the monitoring system, and alerted the team about any detected incident. It also involved knowing the medication and the type of milk that the infants were receiving. Parents who cleaned their infants properly according to their corrected age were considered autonomous in cleanliness. Autonomy in the prevention of health care–associated infections involved the removal of bracelets or watches before contact with the infants, the use of hand sanitizer before touching them, and reminding professionals to use hand sanitizer as well. Finally, autonomy in participation in clinical rounds was achieved when the parents provided suggestions to the team during clinical rounds.

Comparisons between the 2 time periods were performed using the nonparametric Mann-Whitney *U* test and the chi-square test or Fisher exact test. Comparative analyses were adjusted for gestational age and birth weight.

### Ethical Considerations

The Ethics Committee of the Biomedical Research Institute of the 12 de Octubre University Hospital approved the project (21/123). All participants were sent an information document. No compensation was given to families; they voluntarily participated in the care of their children.

## Results

During the study period, a total of 159 VLBW newborn infants were admitted to the neonatal unit. The parents of 107 VLBW newborn infants recorded the dates of complete autonomy in the areas of care at discharge on their road maps. Four families were excluded because of a significant language barrier, and 6 families were excluded because of the death of the newborn in the first month of life. Overall, 54 (72%) out of 75 families were recruited during period 1, and 43 (51%) out of 84 families were recruited during period 2. No statistical differences were found between the infants in periods 1 and 2 (*P*>.50).

The median gestational age and birth weight were 28.7 (IQR 26.8‐31) weeks and 1080 (IQR 850‐1270) g in period 1, respectively. In period 2, they were 27.8 (IQR 26.7‐30.4) weeks and 1000 (800‐1200) g, respectively. The number of days required by the parents of VLBW newborn infants to be autonomous in the 11 care areas included in this study is described and compared in Table 2. It should be noted that the prevention of health care–associated infection was an area of care where health care providers and parents required fewer days to be autonomous (median 6, IQR 3‐12 d), and feeding required more days (median 44.5, IQR 37‐62 d). Additionally, different behaviors can be highlighted when the numbers of days needed to be autonomous were compared between periods 1 and 2. Thus, the number of days required to be autonomous in period 2 decreased for participation in clinical rounds (median 10.5, IQR 5‐20 vs 7, IQR 4‐10.5 d; *P*<.001). In contrast, the time required in period 2 was significantly increased in 3 areas of care: kangaroo mother care (*P*=.02), diaper change (*P*=.04), and prevention of health care–associated infections (*P*<.001).

**Table 2.** The time required by parents of very low–birth weight newborn infants to acquire the highest degree of autonomy in the 11 care areas of the Cuídame program during the implementation.

Area of care	Days, median (IQR)		*P* value	*P* value adjusted for birth weight and gestational age
	Period 1: from September 1, 2020, to July 15, 2021 (n=54)	Period 2: from July 15, 2021, to May 31, 2022 (n=43)		
Kangaroo mother care	14 (7‐23)	21 (10‐31)	*.04*	*.02*
Feeding	53.5 (34-68)	44.5 (37-62)	.39	*.049*
Observation of neurobehavior	18 (9‐33)	11 (7‐16)	*.04*	*.049*
Handling, posture, and contact	16.5 (7-36)	22.5 (11-37)	.24	.17
Diaper change	9.5 (4-20)	14.5 (9-32)	*.01*	*.04*
Stress and pain prevention	13 (6‐26)	12 (7‐26)	*<.001*	.61
Prevention of sensory deprivation	6.5 (2.5-16)	7 (4.5-9)	.35	.82
Patient safety	9 (4-17)	9 (5-19)	.90	.90
Cleanliness	15.5 (7.5-39)	23.5 (13-37)	.25	.07
Prevention of health care–associated infections	1 (1-2)	6 (3-12)	*<.001*	*<.001*
Participation in clinical rounds	10.5 (5-20)	7 (4-10.5)	*.049*	*<.001*

## Discussion

Our study revealed that the implementation of the Cuídame program changed the culture of care in our neonatal unit, resulting in the autonomy of most families in caring for their newborn infants under the supervision of a health care professional during their admission. The prevention of health care–associated infections was the first area where parents achieved the autonomy, and feeding was the last one.

The implementation of a program aimed at achieving parental autonomy in the care of newborn infants does not affect every area of care in the same way. In our case, the time required by parents of VLBW newborn infants to be autonomous in kangaroo mother care, prevention of health care–associated infections, and diaper change increased in the second period. One possible reason for this difference is that health care providers became more demanding during the implementation of the program.

Some limitations should be considered, such as how the COVID-19 epidemic could have acted as a barrier to the implementation of the Cuídame program.

Based on our results, it can be concluded that some areas of care could have different behaviors during the implementation of a training program aimed at achieving parental autonomy. The number of days required by parents to achieve autonomy in kangaroo mother care, diaper change, and the prevention of health care–associated infections increased in our case, even though the team was more experienced. This leads us to believe that achieving parental autonomy is a complex process that depends on several factors in the implementation of a training program.

## Abbreviations

EFCNI: European Foundation for the Care of Newborn Infants
VLBW: very low–birth weight
